# 1-(2-Carb­oxy­eth­yl)-5-ethyl-2-methyl­pyridinium chloride

**DOI:** 10.1107/S1600536812038809

**Published:** 2012-09-15

**Authors:** V. Sabari, G. Kalaiselvi, S. Balasubramanian, S. Aravindhan

**Affiliations:** a Department of Physics, Presidency College, Chennai 600 005, India; b Department of Inorganic Chemistry, University of Madras, Chennai 600 025, India

## Abstract

In the crystal structure of the title salt, C_11_H_16_NO_2_^+^·Cl^−^, the cations and anions are linked by O—H⋯Cl hydrogen bonds. The structure is further stabilized by weak C—H⋯Cl hydrogen bonds.

## Related literature

For the biological activity of 4-amino­pyridine, see: Judge & Bever (2006 ▶); Schwid *et al.* (1997 ▶); Strupp *et al.* (2004 ▶). For related structures, see: Anderson *et al.* (2005 ▶); Fun *et al.* (2009 ▶).
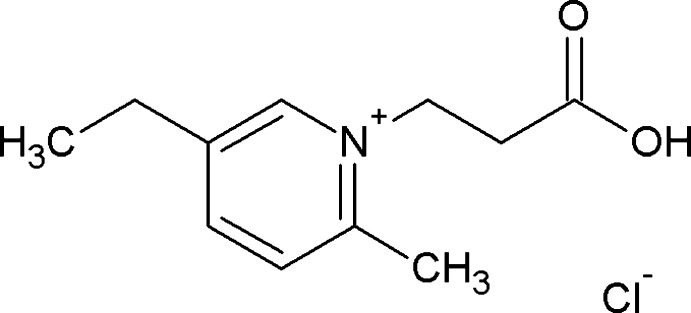


## Experimental

### 

#### Crystal data

C_11_H_16_NO_2_^+^·Cl^−^
*M* *_r_* = 229.70
Triclinic, 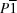

*a* = 7.5013 (4) Å
*b* = 9.0509 (5) Å
*c* = 9.3452 (5) Å
α = 75.253 (2)°
β = 80.985 (2)°
γ = 72.047 (2)°
*V* = 581.59 (5) Å^3^
*Z* = 2
Mo *K*α radiation
μ = 0.31 mm^−1^
*T* = 293 K
0.32 × 0.20 × 0.10 mm

#### Data collection

Bruker APEXII CCD area-detector diffractometer
Absorption correction: multi-scan (*SADABS*; Bruker, 2008 ▶) *T*_min_ = 0.972, *T*_max_ = 0.992
11668 measured reflections
2772 independent reflections
2363 reflections with *I* > 2σ(*I*)
*R*_int_ = 0.026

#### Refinement

*R*[*F* ^2^ > 2σ(*F* ^2^)] = 0.033
*wR*(*F* ^2^) = 0.102
*S* = 0.83
2772 reflections
142 parameters
H atoms treated by a mixture of independent and constrained refinement
Δρ_max_ = 0.23 e Å^−3^
Δρ_min_ = −0.19 e Å^−3^

### 

Data collection: *APEX2* (Bruker, 2008 ▶); cell refinement: *SAINT* (Bruker, 2008 ▶); data reduction: *SAINT*; program(s) used to solve structure: *SHELXS97* (Sheldrick, 2008 ▶); program(s) used to refine structure: *SHELXL97* (Sheldrick, 2008 ▶); molecular graphics: *ORTEP-3* (Farrugia, 1997 ▶); software used to prepare material for publication: *SHELXL97* and *PLATON* (Spek, 2009 ▶).

## Supplementary Material

Crystal structure: contains datablock(s) I, global. DOI: 10.1107/S1600536812038809/bt6827sup1.cif

Structure factors: contains datablock(s) I. DOI: 10.1107/S1600536812038809/bt6827Isup2.hkl

Supplementary material file. DOI: 10.1107/S1600536812038809/bt6827Isup3.cml

Additional supplementary materials:  crystallographic information; 3D view; checkCIF report

## Figures and Tables

**Table 1:** Hydrogen-bond geometry (Å, °)

*D*—H⋯*A*	*D*—H	H⋯*A*	*D*⋯*A*	*D*—H⋯*A*
O2—H2*A*⋯Cl1	0.92 (3)	2.06 (3)	2.9749 (12)	170 (2)
C2—H2⋯Cl1^i^	0.93	2.72	3.6249 (14)	166
C6—H6*A*⋯Cl1^ii^	0.97	2.68	3.6261 (14)	166
C11—H11*A*⋯Cl1^iii^	0.96	2.79	3.7410 (16)	170

Symmetry codes: (i) 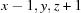; (ii) 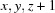; (iii) 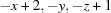.

**Fig. 1.**
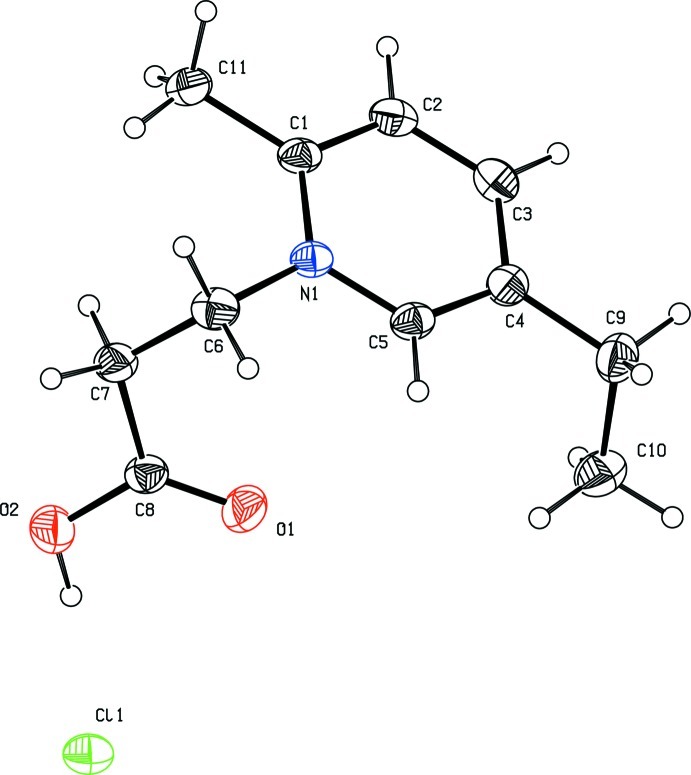
View of one molecule of the title compound showing the atom-labelling scheme. Displacement ellipsoids are drawn at the 30% probability level (arbitrary spheres for the H atoms).

## supplementary crystallographic information

### Comment

4-Aminopyridine (Fampridine) is used clinically in Lambert-Eaton myasthenic syndrome and multiple sclerosis because by blocking potassium channels it prolongs action potentials thereby increasing transmitter release at the neuromuscular junction (Judge & Bever *et al.*, 2006; Schwid *et al.*, 1997; Strupp *et al.*, 2004).

In the title compound (Fig. 1), the bond lengths and angles have normal values. The asymmetric unit is composed of one 1-(2-carboxy ethyl) 5-ethyl 2-methyl pyridinium cation and one Cl^-^ anion. The C1—N1—C5 angle in the pyridinium ring is widened to 121.20 (15) °, compared to 115.25 (13)° in 4-aminopyridine (Anderson *et al.*, 2005) and 120.7 (2)°, in Aminopyridinium (Fun *et al.*, 2009). In the crystal structure, anions and cations are connected by O—H···Cl and C—H···Cl hydrogen bonds.

### Experimental

1g (8.3mmol) of freshly distilled 5-ethyl 2-methyl pyridine was dissolved in 10 ml of THF at -10°C under nitrogen atmosphere. To the above solution, 0.8 ml (8.0 mmol) of acrylic acid in 10 ml of THF was added drop wise with continuous stirring. After stirring for 20 min in an ice bath, 0.5 mL of HCl was added and stirred for 24 h. White solid formed after the completion of the reaction and the solid was filtered, washed with THF and dried in vacuum. The product was recrystallized from methanol Yield: 1.52g (80%)

### Refinement

All H atoms on carbons were positioned geometrically with C—H distances ranging from 0.95 to 1.00 Å and refined as riding on their parent atoms, with *U*_iso_(H) = 1.2Ueq(C) or *U*_iso_(H) = 1.5Ueq(C_methyl_) The hydroxyl H atom was freely refined.

### Crystal data

**Table:**

C_11_H_16_NO_2_^+^·Cl^−^	*Z* = 2
*M**_r_* = 229.70	*F*(000) = 244
Triclinic, *P*1	*D*_x_ = 1.312 Mg m^−^^3^
Hall symbol: -P 1	Mo *K*α radiation, λ = 0.71073 Å
*a* = 7.5013 (4) Å	Cell parameters from 5710 reflections
*b* = 9.0509 (5) Å	θ = 1.8–28.5°
*c* = 9.3452 (5) Å	µ = 0.31 mm^−^^1^
α = 75.253 (2)°	*T* = 293 K
β = 80.985 (2)°	Triclinic, colourless
γ = 72.047 (2)°	0.32 × 0.20 × 0.10 mm
*V* = 581.59 (5) Å^3^	

### Data collection

**Table:**

Bruker APEXII CCD area-detector diffractometer	2772 independent reflections
Radiation source: fine-focus sealed tube	2363 reflections with *I* > 2σ(*I*)
Graphite monochromator	*R*_int_ = 0.026
ω and φ scans	θ_max_ = 27.9°, θ_min_ = 2.3°
Absorption correction: multi-scan (*SADABS*; Bruker, 2008)	*h* = −9→9
*T*_min_ = 0.972, *T*_max_ = 0.992	*k* = −11→11
11668 measured reflections	*l* = −10→12

### Refinement

**Table:**

Refinement on *F*^2^	Secondary atom site location: difference Fourier map
Least-squares matrix: full	Hydrogen site location: inferred from neighbouring sites
*R*[*F*^2^ > 2σ(*F*^2^)] = 0.033	H atoms treated by a mixture of independent and constrained refinement
*wR*(*F*^2^) = 0.102	*w* = 1/[σ^2^(*F*_o_^2^) + (0.0665*P*)^2^ + 0.2215*P*] where *P* = (*F*_o_^2^ + 2*F*_c_^2^)/3
*S* = 0.83	(Δ/σ)_max_ < 0.001
2772 reflections	Δρ_max_ = 0.23 e Å^−^^3^
142 parameters	Δρ_min_ = −0.19 e Å^−^^3^
0 restraints	Extinction correction: *SHELXL97* (Sheldrick, 2008), Fc^*^=kFc[1+0.001xFc^2^λ^3^/sin(2θ)]^-1/4^
Primary atom site location: structure-invariant direct methods	Extinction coefficient: 0.034 (6)

### Special details

**Table:**

Geometry. All e.s.d.'s (except the e.s.d. in the dihedral angle between two l.s. planes) are estimated using the full covariance matrix. The cell e.s.d.'s are taken into account individually in the estimation of e.s.d.'s in distances, angles and torsion angles; correlations between e.s.d.'s in cell parameters are only used when they are defined by crystal symmetry. An approximate (isotropic) treatment of cell e.s.d.'s is used for estimating e.s.d.'s involving l.s. planes.
Refinement. Refinement of *F*^2^ against ALL reflections. The weighted *R*-factor *wR* and goodness of fit *S* are based on *F*^2^, conventional *R*-factors *R* are based on *F*, with *F* set to zero for negative *F*^2^. The threshold expression of *F*^2^ > σ(*F*^2^) is used only for calculating *R*-factors(gt) *etc*. and is not relevant to the choice of reflections for refinement. *R*-factors based on *F*^2^ are statistically about twice as large as those based on *F*, and *R*- factors based on ALL data will be even larger.

### Fractional atomic coordinates and isotropic or equivalent isotropic displacement parameters (Å^2^)

**Table:**

	*x*	*y*	*z*	*U*_iso_*/*U*_eq_	
O2	1.11724 (18)	0.12433 (14)	0.49990 (13)	0.0607 (3)	
Cl1	1.13273 (5)	0.19233 (4)	0.17024 (4)	0.04786 (14)	
N1	0.73265 (15)	0.39471 (13)	0.81065 (11)	0.0344 (2)	
C1	0.60912 (19)	0.31985 (15)	0.89780 (13)	0.0373 (3)	
C8	1.01152 (18)	0.23770 (16)	0.56489 (14)	0.0388 (3)	
C5	0.67204 (19)	0.53934 (15)	0.72045 (13)	0.0374 (3)	
H5	0.7607	0.5877	0.6639	0.045*	
O1	0.92281 (18)	0.36464 (13)	0.49974 (11)	0.0570 (3)	
C4	0.4848 (2)	0.61779 (16)	0.70897 (14)	0.0390 (3)	
C11	0.6787 (2)	0.16261 (18)	1.00116 (16)	0.0503 (4)	
H11A	0.7271	0.0810	0.9453	0.075*	
H11B	0.5770	0.1396	1.0707	0.075*	
H11C	0.7768	0.1662	1.0539	0.075*	
C3	0.3566 (2)	0.54196 (18)	0.79512 (16)	0.0441 (3)	
H3	0.2282	0.5900	0.7897	0.053*	
C2	0.4195 (2)	0.39556 (17)	0.88856 (15)	0.0430 (3)	
H2	0.3323	0.3465	0.9468	0.052*	
C6	0.93976 (19)	0.32296 (19)	0.81201 (15)	0.0436 (3)	
H6A	0.9709	0.2834	0.9143	0.052*	
H6B	1.0014	0.4055	0.7671	0.052*	
C7	1.0172 (2)	0.18822 (18)	0.73100 (15)	0.0448 (3)	
H7B	1.1466	0.1356	0.7534	0.054*	
H7A	0.9463	0.1110	0.7692	0.054*	
C9	0.4262 (2)	0.77820 (17)	0.60617 (17)	0.0510 (4)	
H9B	0.4899	0.8477	0.6273	0.061*	
H9A	0.2920	0.8242	0.6246	0.061*	
C10	0.4713 (3)	0.7697 (2)	0.44375 (17)	0.0566 (4)	
H10A	0.4327	0.8749	0.3825	0.085*	
H10C	0.4054	0.7038	0.4214	0.085*	
H10B	0.6042	0.7252	0.4247	0.085*	
H2A	1.108 (3)	0.153 (3)	0.399 (3)	0.099 (8)*	

### Atomic displacement parameters (Å^2^)

**Table:**

	*U*^11^	*U*^22^	*U*^33^	*U*^12^	*U*^13^	*U*^23^
O2	0.0687 (7)	0.0570 (7)	0.0404 (6)	0.0055 (6)	−0.0043 (5)	−0.0115 (5)
Cl1	0.0537 (2)	0.0563 (2)	0.03510 (19)	−0.01908 (17)	0.00042 (14)	−0.01085 (14)
N1	0.0393 (5)	0.0394 (6)	0.0274 (5)	−0.0143 (4)	−0.0016 (4)	−0.0091 (4)
C1	0.0487 (7)	0.0390 (7)	0.0276 (5)	−0.0171 (6)	0.0032 (5)	−0.0114 (5)
C8	0.0357 (6)	0.0436 (7)	0.0364 (6)	−0.0143 (5)	−0.0016 (5)	−0.0048 (5)
C5	0.0470 (7)	0.0387 (7)	0.0306 (6)	−0.0185 (5)	−0.0004 (5)	−0.0088 (5)
O1	0.0768 (8)	0.0457 (6)	0.0376 (5)	−0.0042 (5)	−0.0076 (5)	−0.0044 (4)
C4	0.0499 (7)	0.0368 (6)	0.0329 (6)	−0.0116 (5)	−0.0040 (5)	−0.0124 (5)
C11	0.0660 (9)	0.0424 (8)	0.0364 (7)	−0.0154 (7)	0.0070 (6)	−0.0044 (6)
C3	0.0407 (7)	0.0496 (8)	0.0443 (7)	−0.0111 (6)	−0.0002 (6)	−0.0185 (6)
C2	0.0454 (7)	0.0486 (8)	0.0395 (7)	−0.0211 (6)	0.0081 (6)	−0.0148 (6)
C6	0.0391 (7)	0.0578 (8)	0.0350 (6)	−0.0148 (6)	−0.0088 (5)	−0.0070 (6)
C7	0.0382 (7)	0.0514 (8)	0.0350 (6)	−0.0061 (6)	−0.0022 (5)	−0.0010 (6)
C9	0.0661 (10)	0.0376 (7)	0.0460 (8)	−0.0078 (7)	−0.0099 (7)	−0.0087 (6)
C10	0.0693 (10)	0.0565 (9)	0.0428 (8)	−0.0199 (8)	−0.0084 (7)	−0.0039 (7)

### Geometric parameters (Å, º)

**Table:**

O2—C8	1.3097 (18)	C11—H11C	0.9600
O2—H2A	0.92 (3)	C3—C2	1.377 (2)
N1—C5	1.3499 (17)	C3—H3	0.9300
N1—C1	1.3604 (16)	C2—H2	0.9300
N1—C6	1.4885 (17)	C6—C7	1.514 (2)
C1—C2	1.381 (2)	C6—H6A	0.9700
C1—C11	1.4938 (19)	C6—H6B	0.9700
C8—O1	1.1971 (17)	C7—H7B	0.9700
C8—C7	1.5058 (19)	C7—H7A	0.9700
C5—C4	1.372 (2)	C9—C10	1.519 (2)
C5—H5	0.9300	C9—H9B	0.9700
C4—C3	1.388 (2)	C9—H9A	0.9700
C4—C9	1.5009 (19)	C10—H10A	0.9600
C11—H11A	0.9600	C10—H10C	0.9600
C11—H11B	0.9600	C10—H10B	0.9600
			
C8—O2—H2A	110.8 (16)	C3—C2—H2	119.3
C5—N1—C1	121.15 (11)	C1—C2—H2	119.3
C5—N1—C6	116.99 (11)	N1—C6—C7	114.17 (11)
C1—N1—C6	121.85 (11)	N1—C6—H6A	108.7
N1—C1—C2	117.68 (12)	C7—C6—H6A	108.7
N1—C1—C11	120.43 (13)	N1—C6—H6B	108.7
C2—C1—C11	121.88 (12)	C7—C6—H6B	108.7
O1—C8—O2	123.99 (13)	H6A—C6—H6B	107.6
O1—C8—C7	124.60 (13)	C8—C7—C6	114.89 (12)
O2—C8—C7	111.41 (12)	C8—C7—H7B	108.5
N1—C5—C4	122.58 (12)	C6—C7—H7B	108.5
N1—C5—H5	118.7	C8—C7—H7A	108.5
C4—C5—H5	118.7	C6—C7—H7A	108.5
C5—C4—C3	117.08 (13)	H7B—C7—H7A	107.5
C5—C4—C9	120.07 (13)	C4—C9—C10	112.48 (12)
C3—C4—C9	122.85 (14)	C4—C9—H9B	109.1
C1—C11—H11A	109.5	C10—C9—H9B	109.1
C1—C11—H11B	109.5	C4—C9—H9A	109.1
H11A—C11—H11B	109.5	C10—C9—H9A	109.1
C1—C11—H11C	109.5	H9B—C9—H9A	107.8
H11A—C11—H11C	109.5	C9—C10—H10A	109.5
H11B—C11—H11C	109.5	C9—C10—H10C	109.5
C2—C3—C4	119.99 (13)	H10A—C10—H10C	109.5
C2—C3—H3	120.0	C9—C10—H10B	109.5
C4—C3—H3	120.0	H10A—C10—H10B	109.5
C3—C2—C1	121.50 (12)	H10C—C10—H10B	109.5
			
C5—N1—C1—C2	1.26 (17)	C4—C3—C2—C1	−0.9 (2)
C6—N1—C1—C2	−179.81 (11)	N1—C1—C2—C3	−0.25 (19)
C5—N1—C1—C11	−177.69 (11)	C11—C1—C2—C3	178.69 (13)
C6—N1—C1—C11	1.24 (18)	C5—N1—C6—C7	−105.73 (14)
C1—N1—C5—C4	−1.18 (18)	C1—N1—C6—C7	75.30 (15)
C6—N1—C5—C4	179.84 (11)	O1—C8—C7—C6	−14.0 (2)
N1—C5—C4—C3	0.01 (19)	O2—C8—C7—C6	165.58 (13)
N1—C5—C4—C9	−179.76 (11)	N1—C6—C7—C8	69.15 (16)
C5—C4—C3—C2	0.99 (19)	C5—C4—C9—C10	68.80 (18)
C9—C4—C3—C2	−179.24 (13)	C3—C4—C9—C10	−110.95 (16)

### Hydrogen-bond geometry (Å, º)

**Table:**

*D*—H···*A*	*D*—H	H···*A*	*D*···*A*	*D*—H···*A*
C2—H2···Cl1^i^	0.93	2.72	3.6249 (14)	166
C6—H6*A*···Cl1^ii^	0.97	2.68	3.6261 (14)	166
O2—H2*A*···Cl1	0.92 (3)	2.06 (3)	2.9749 (12)	170 (2)
C11—H11*A*···Cl1^iii^	0.96	2.79	3.7410 (16)	170

Symmetry codes: (i) *x*−1, *y*, *z*+1; (ii) *x*, *y*, *z*+1; (iii) −*x*+2, −*y*, −*z*+1.
